# Melatonin Prevents Oxidative Stress-Induced Mitochondrial Dysfunction and Apoptosis in High Glucose-Treated Schwann Cells via Upregulation of Bcl2, NF-κB, mTOR, Wnt Signalling Pathways

**DOI:** 10.3390/antiox8070198

**Published:** 2019-06-26

**Authors:** Yee Lian Tiong, Khuen Yen Ng, Rhun Yian Koh, Gnanajothy Ponnudurai, Soi Moi Chye

**Affiliations:** 1 School of Postgraduate Studies, International Medical University, Kuala Lumpur 57000, Malaysia; 2 School of Pharmacy, Monash University Malaysia, Selangor 47500, Malaysia; 3 School of Health Science, International Medical University, Kuala Lumpur 57000, Malaysia; 4 School of Medicine, International Medical University, Kuala Lumpur 57000, Malaysia

**Keywords:** melatonin, apoptosis, reactive oxygen species, mitochondrial, Bcl-2, NF-κB, mTOR, Wnt

## Abstract

Neuropathy is a complication that affects more than 50% of long-standing diabetic patients. One of the causes of diabetes neuropathy (DN) is the apoptosis of Schwann cells due to prolonged exposure to high glucose and build-up of oxidative stress. Melatonin is a hormone that has a known antioxidant property. In this study, we investigated the protective effect of melatonin on high glucose-induced Schwann cells’ apoptosis. Our results revealed that high glucose promoted apoptosis via mitochondrial-related oxidative stress and downregulated Bcl-2 family proteins in Schwann cells. In this signalling pathway, Bcl-2, Bcl-XL and Mcl-1 proteins were down-regulated while p-BAD and Puma proteins were up-regulated by high glucose treatment. Besides, we also proved that high glucose promoted apoptosis in Schwann cells through decreasing the p-NF-κB in the NF-κB signalling pathway. Key regulators of mTOR signalling pathway such as p-mTOR, Rictor and Raptor were also down-regulated after high glucose treatment. Additionally, high glucose treatment also decreased the Wnt signalling pathway downstream proteins (Wnt 5a/b, p-Lrp6 and Axin). Our results showed that melatonin treatment significantly inhibited high glucose-induced ROS generation, restored mitochondrial membrane potential and inhibited high glucose-induced apoptosis in Schwann cells. Furthermore, melatonin reversed the alterations of protein expression caused by high glucose treatment. Our results concluded that melatonin alleviates high glucose-induced apoptosis in Schwann cells through mitigating mitochondrial-related oxidative stress and the alterations of Bcl-2, NF-κB, mTOR and Wnt signalling pathways.

## 1. Introduction

Approximately 50% of patients with longstanding diabetes mellitus (DM) eventually develop DN [1]. Growing evidence suggests that the pathophysiology of diabetes neuropathy (DN) is constituted by a number of interwoven pathways including increased flux of glucose to the polyol pathway, increased hexosamine shunt, aldose reductase activation, decrease in nerve myo-inositol content, formation of advanced glycation end product (AGE), impaired neurotrophic support and activation of protein kinase C (PKC). All these events result in the overproduction of reactive oxygen species (ROS) which ultimately damages Schwann cells resulting in DN [2,3,4].

ROS has been reported to be associated with impaired mitochondrial function. Elevation of ROS promotes outer mitochondrial membrane permeabilization through opening of a mitochondrial permeability transition pore that allows the flow of molecules <1.5kDa. The resulting depolarisation is followed by collapse of the mitochondrial membrane potential (MMP) leading to the release of pro-apoptotic factors such as cytochrome c, caspases, and subsequently promotes apoptosis [5,6,7,8,9].

NF-κB transcription factor is a critical regulator of apoptosis. NF-κB-dependent transcription is closely coordinated with other signalling pathways through its downstream I kappa B (IκB) and IκB kinase (IKK) proteins. For example, crosstalk between NF-κB and mTOR pathway plays an important role in inducing apoptosis. It is known that inhibition of mTOR and/or regulatory associated protein of mTOR (Raptor) induces apoptosis by blocking NF-κB activation and suppressing NF-κB-dependent gene expression [9]. Apart from that, NF-κB also plays an important role in Wnt-induced apoptosis signalling pathway [10].

Melatonin is an indole secreted by the pineal gland but also present in the diet [11]. The most common physiological effect of melatonin is coordinating circadian activity. It serves as a chronobiotic factor to maintain circadian rhythm amplitudes and reinforce oscillations of the biological clock [12,13]. Secretion of melatonin is tightly regulated by light, thus disruption usually occurs in individuals deprived of light such as travellers across time zone and night-shift workers. Moreover, melatonin has antioxidant property, specificity, electron-rich aromatic indole ring of melatonin is a potent electron donor that can significantly reduce oxidative stress, and upregulating the expressions of antioxidant enzymes through melatonin receptors (MT1 and MT2) [14,15]. Apart from that, melatonin has anti-apoptotic property and exerts anti-apoptotic actions through preventing cytochrome c release [16], reducing caspase-1 and caspase-3 activation, increasing anti-apoptotic proteins (Bcl-2, Bcl-xL) expression [17], and decreasing pro-apoptotic proteins (Bad, Bax) expression [18]. However, the protective mechanism of melatonin on high glucose-induced Schwann cell apoptosis have not yet been fully established. Thus, the objective of this study is to investigate the effects of melatonin on high glucose-induced Schwann cell apoptosis through of Bcl-2, NF-κB, mTOR and Wnt signalling pathways.

## 2. Materials and Methods

### 2.1. Materials

Dulbecco’s modified Eagle’s medium (DMEM), foetal bovine serum (FBS), and trypsin-EDTA were purchased from GIBCO Laboratories (Grand Island, NY, USA). Melatonin, glucose, dimethylsulfoxide (DMSO), Triton X-100, NP-40, ethylenediaminetetraacetic acid (EDTA), Tris-HCl, phosphate buffered saline (PBS), horseradish peroxidase-conjugated secondary antibodies, dithiothreitol (DTT), sodium dodecyl sulphate (SDS), ammonium acetate, Tris-borate-EDTA buffer, Bradford reagent, and phenylmethyl sulfonyl fluoride were purchased from Sigma Chemical Co. (St Louis, MO, USA). An Annexin-V-Fluos staining kit was purchased from Roche Diagnosis GmbH (Penzberg, Germany). Proteinase K, ribonuclease A (RNase A) and antibodies were obtained from BD Biosciences Pharmingen (San Diego, CA, USA). ECL-Plus kits and polyvinylidene difluoride (PVDF) membranes were obtained from Amersham Biosciences (Pittsburgh, PA, USA). All chemicals were of the highest grade commercially available.

### 2.2. Cell Culture and Treatment

RT4-D6P2T Schwann cells were obtained from the American Type Culture Collection (Rockville, MD, USA). The cells were cultured with DMEM supplemented with 10% FBS and maintained in the incubator at 37 °C with humidified atmosphere containing 5% CO_2_. The stock solution of melatonin (215.275 µM) was dissolved in absolute ethanol and different concentrations were prepared in the culture medium. The stock solution of glucose (1 M) was dissolved in DMEM medium and different concentrations were prepared in the culture medium.

### 2.3. Measurement of Cell Viability

Glucose and melatonin concentrations were selected according to the publications of Delaney et al., (2001) and Li et al., (2018) [19,20]. The Schwann cells were treated with 100 mM of glucose with or without melatonin (0.5, 1, 5 and 10 μM) for 24 h. Cell viability was measured by 3-(4,5-dimethylthiazol-2-yl)-2,5-diphenyltetrazolium bromide (MTT) assay. Twenty μL of MTT solution was added into each well and then incubated for an additional 3 h. After centrifugation, the supernatant was removed and 100 µL of DMSO was then added to dissolve the purple crystals of formazan. Samples were then read at 570 nm using a spectrophotometer. Results were collected from three replicates in three independent experiments. The results were reported as the percentage of cell growth in the tested group versus the control group.

### 2.4. Measurement of Reactive Oxygen Species (ROS)

The Schwann cells were cultured in 96 wells plate, then treated with 100 mM of glucose with or without melatonin (0.5, 1, 5 and 10 μM) for 24 h. The cells were stained with 10 µM of dichloro-dihydro-fluorescein diacetate (DCFH-DA) for 30 min. The cells were then examined under a fluorescence microscope. Fluorescence intensity in the cells was detected using a fluorescence plate reader at an excitation wavelength of 488 nm and an emission wavelength of 525 nm.

### 2.5. Measurement of Mitochondrial Trans-Membrane Potential (ΔΨm)

Change in ΔΨm was examined using MitoScreen kit (BD PharmingenTM). The Schwann cells were cultured in 60 mm culture dishes, then treated with 100 mM of glucose with or without melatonin (0.5, 1, 5 and 10 μM) for 24 h. Both floating and adherent cells were collected, washed with PBS and then centrifuged at 1500 rpm for 5 min. The cells were then resuspended in 500 µL of working solution consisting of 5,5′,6,6′-tetrachloro-1,1′,3,3′-tetraethylbenzimidazolcarbocyanine iodide (JC-1) dye and incubated at 37 °C for 15 min in dark. Finally, ΔΨm was analysed using a Becton-Dickinson FACS-Calibur flow cytometer.

### 2.6. Measurement of Cell Apoptosis

The Schwann cells were cultured in 60 mm tissue culture dishes, then treated with 100 mM of glucose with or without melatonin (0.5, 1, 5 and 10 μM) for 24 h. After the incubation, floating as well as adherent cells were collected, trypsinised and then centrifuged at 1000× *g* for 5 min. Pelleted cells were washed with PBS. Thereafter, cells were centrifuged at 1000× *g* for another 5 min and resuspended in 100 μL of Annexin-V-Fluos and PI labelling solution for 10 min. The stained cells were analysed by flow cytometer, where the fluorescence emission was measured at 530 nm (Alexa Fluor 488). The percentage of cell undergoing apoptosis was calculated using the CellQuest software (BD Biosciences, Franklin Lakes, NJ, USA).

### 2.7. Measurement of Protein Expression

The Schwann cells were cultured in 60 mm tissue culture dishes, then treated with 100 mM of glucose with or without melatonin (0.5, 1, 5 and 10 μM) for 24 h. Then, adherent and floating cells were collected and homogenised in a lysis buffer (10 mM Tris–HCl, pH 8; 0.32 mM sucrose; 5 mM EDTA; 2 mM DTT; 1 mM phenylmethyl sulfonyl fluoride; and 1% Triton X-100). After centrifugation, the supernatant was collected and assayed for protein concentration using the Bradford method. An equal amount of protein per sample was subjected to 10% SDS-polyacrylamide gel electrophoresis. After electrophoresis, the proteins were transferred to PVDF membranes by electroblotting and incubated with diluted primary antibodies for 1 h at 25 °C. After washing with PBS, the membranes were incubated with diluted horseradish peroxidase (HRP)-conjugated secondary antibodies for 30 min at 25 °C. The protein bands were detected by chemiluminescence using an ECL-Plus kit (Amersham Biosciences, Pittsburgh, PA, USA) and quantified using Image Lab software (version 4.1, Bio-Rad Laboratories, CA, USA).

### 2.8. Statistical Analysis

The results were expressed as the mean ± standard deviation (SD) from at least three independent experiments. Statistical analysis was performed using the analysis of variance (ANOVA) test. The significance level was set as *p* < 0.05. The error bars denote SD.

## 3. Results

### 3.1. Effects of Melatonin on the Cell Viability of High Glucose-Treated Schwann Cells

**Figure 1:**
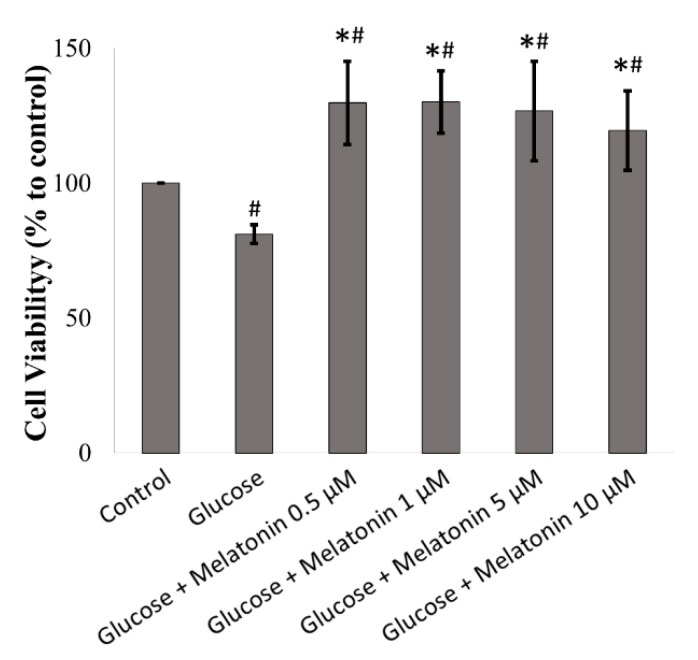
Effects of melatonin on the cell viability of high glucose-treated Schwann cells. The Schwann cells were treated with 100 mM of glucose with or without melatonin (0.5, 1, 5 and 10 μM) for 24 h and cell viability was measured by MTT assay. Each bar represents mean ± standard deviation (SD) of three independent experiments. * *p* < 0.05 significant different vs 100 mM glucose; # *p* < 0.05 vs control.

The effects of melatonin on cell viability of high glucose-treated Schwann cell was measured by MTT assay. As shown in Figure 1, after treatment with 100 mM of glucose for 24 h, glucose significantly decreased cell viability to 81% as compared to control cells. However, melatonin treatment (0.5, 1, 5 and 10 μM) prevented glucose-induced cell death and increased cell viability under high glucose condition. Apart from that, under microscopic examination, the morphology of Schwann cells appeared oval, spindle-shaped or bipolar. After being treated with high glucose, there were few changes in cell morphology, and most of the cells remained oval or spindle-shaped, with a small number of cells becoming rounded and stopping proliferation.

### 3.2. Effect of Melatonin on ROS Generation of High Glucose-Treated Schwann Cells

**Figure 2:**
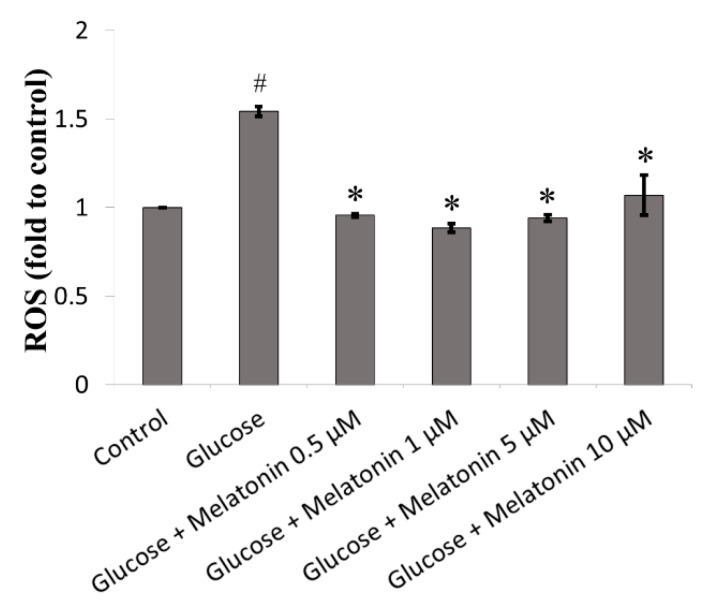
Effects of melatonin on reactive oxygen species (ROS) generation of high glucose-treated Schwann cells. The Schwann cells were treated with 100 mM of glucose with or without melatonin (0.5, 1, 5 and 10 μM) for 24 h and ROS generation was measured by dichloro-dihydro-fluorescein diacetate (DCFH-DA) staining. Each bar represents mean ± SD of three independent experiments. * *p* < 0.05 significant different vs. 100 mM glucose; # *p* < 0.05 vs. control.

The effects of melatonin on ROS generation of high glucose-treated Schwann cells were measured by DCFH-DA staining. ROS level in high glucose-treated Schwann cell was significantly elevated 1.54 fold as compared to control. However, melatonin treatment (0.5, 1, 5 and 10 μM) significantly attenuated high glucose-induced ROS generation in Schwann cells as shown in Figure 2.

### 3.3. Effects of Melatonin on Mitochondrial Membrane Potential (ΔΨm) Changes in High Glucose-Treated Schwann Cells

**Figure 3:**
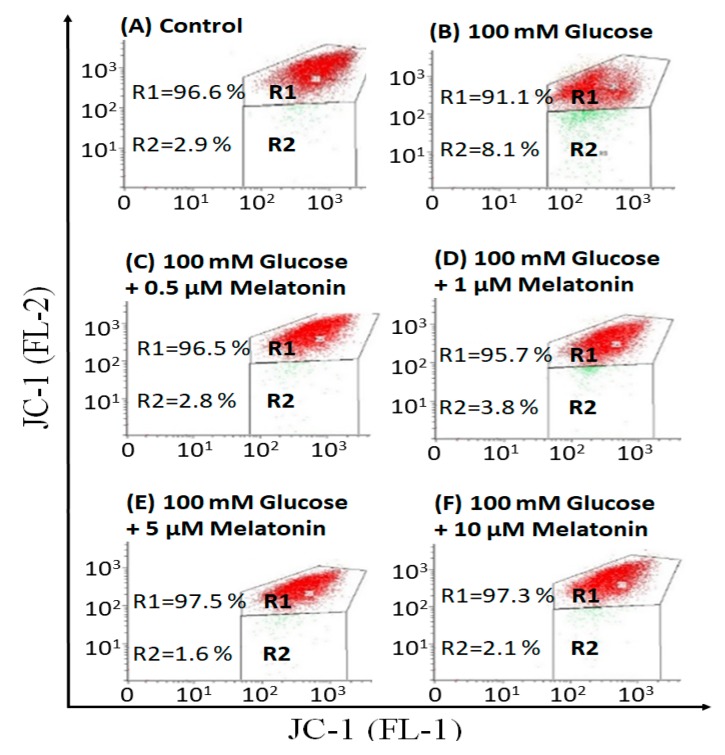
Effects of melatonin on mitochondrial membrane potential (ΔΨm) changes of high glucose-treated Schwann cells. The Schwann cells were treated with 100 mM of glucose with or without melatonin (0.5, 1, 5 and 10 μM) for 24 h and ΔΨm changes was measured by 5,5′,6,6′-tetrachloro-1,1′,3,3′-tetraethylbenzimidazolcarbocyanine iodide (JC-1) staining. **A**–**F** are the representative dot plots of the high glucose-treated cells with or without melatonin. Each data was expressed as mean ± SD of three independent experiments.

The effects of melatonin on ΔΨm changes in high glucose-treated Schwann cells was measured by JC-1 dye staining. In healthy cells with high ΔΨm, JC-1 spontaneously forms complexes known as J-aggregates with intense red fluorescence. On the other hand, in apoptotic or unhealthy cells with low ΔΨm, JC-1 remains in the monomeric form, which shows only green fluorescence. As shown in Figure 3, high glucose-treated Schwann cells significantly decreased ΔΨm when compared with control. However, melatonin treatment (0.5, 1, 5 and 10 μM) increased ΔΨm when compared with high glucose-treated cells.

### 3.4. Effects of Melatonin on High Glucose-Induced Apoptosis in Schwann Cells

**Figure 4:**
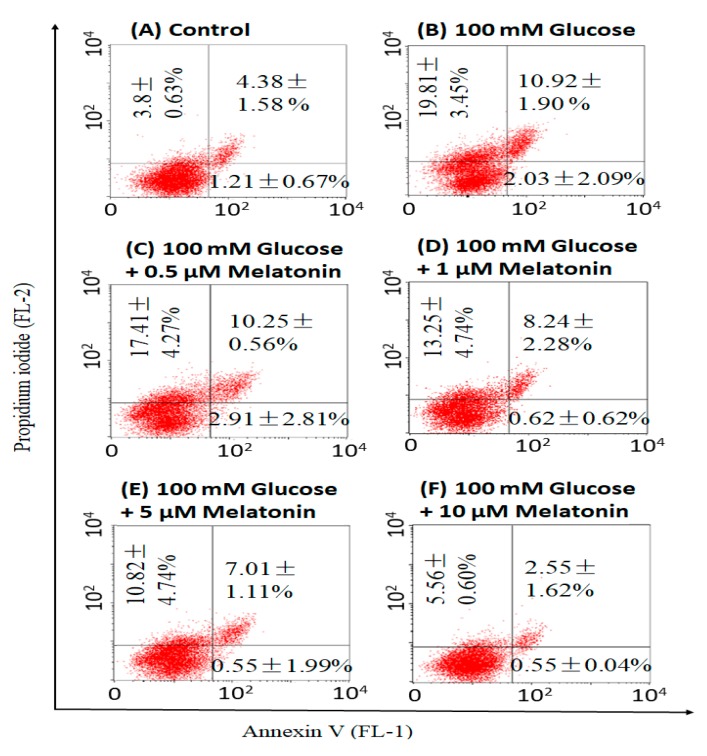
Effects of melatonin on high glucose-induced apoptosis in Schwann cells. The Schwann cells were treated with 100 mM of glucose with or without melatonin (0.5, 1, 5 and 10 μM) for 24 h and apoptosis was measured by Annexin V/propidium iodide staining. **A**–**F** are the representative dot plots of the high glucose-treated cells with or without melatonin. Each data was expressed as mean ± SD of three independent experiments.

The effects of melatonin on incidence of apoptosis in high glucose-treated Schwann cells was measured by Annexin V/propidium iodide staining. Our results showed that high glucose-induced both early and late apoptosis in Schwann cells, approximately 3.1- and 2.5-fold of that in control respectively. Percentage of apoptotic cells (both early and late apoptosis) were comparably less in melatonin with high glucose-treated cells as compared to high glucose-treated cells alone (Figure 4). This indicated that melatonin is effectively reducing apoptosis in high glucose-treated Schwann cells.

### 3.5. Effects of Melatonin on Bcl-2 Family Proteins Expression in High Glucose-Treated Schwann Cells

**Figure 5:**
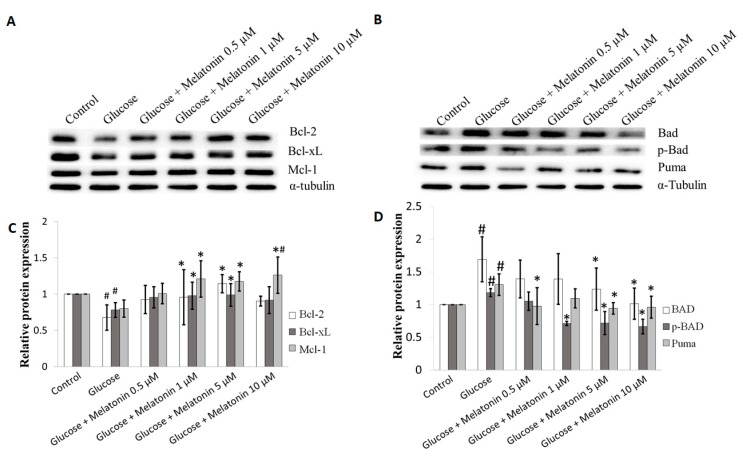
Effects of melatonin on Bcl-2 family proteins expression in high glucose-treated Schwann cells. The Schwann cells were treated with 100 mM of glucose with or without melatonin (0.5, 1, 5 and 10 μM) for 24 h. (**A**,**B**) represent of Bcl-2 family proteins expression were measured by Western blot. Tubulin was used as the housekeeping protein. (**C**,**D**) are the bar charts of proteins expression; each bar represents mean ± SD of three independent experiments. * *p* < 0.05 significant different vs. 100 mM glucose; # *p* < 0.05 vs. control.

The effects of melatonin on Bcl-2 family proteins expression in high glucose-treated Schwann cells was measured by Western blot. Bcl-2 family consists of anti-apoptotic and pro-apoptotic proteins. Anti-apoptotic bcl-2 family proteins, including Bcl-2, Bcl-xL, Bcl-w, Bcl-b, Mcl-1 and A1/BFL1 inhibit apoptosis. In contrast, the pro-apoptotic Bcl-2 family proteins, including Bax, Bak, Bim, Bmf, Bad, Bid, Noxa and Puma promote apoptosis. Bax and Bak are the pro-apoptotic proteins that promote outer mitochondrial membrane permeabilization leading to the release of apoptogenic factor such as cytochrome C to activate apoptosis [21,22]. Our results showed that Bcl-2, Bcl-xL and Mcl-1 proteins expression of pro-survival Bcl-2 family were downregulated after high glucose treatment. However, melatonin treatment (0.5, 1, 5 and 10 μM) seems to attenuate these changes by up-regulating expression of the Bcl-2, Bcl-xL and Mcl-1 proteins and the best effects were obtained at 5 and 10 µM melatonin (Figure 5A,C). In contrast, pro-apoptotic Bcl-2 family proteins expression including BAD, p-BAD and Puma were significantly up-regulated in Schwann cell treated by high glucose. Again, melatonin (0.5, 1, 5 and 10 μM) seems to reverse these changes by down-regulating BAD, p-BAD and Puma proteins expression and the best effects were obtained at 5 and 10 µM melatonin (Figure 5B,D).

### 3.6. Effects of Melatonin on NF-κB Proteins Expression in High Glucose-Treated Schwann Cells

**Figure 6:**
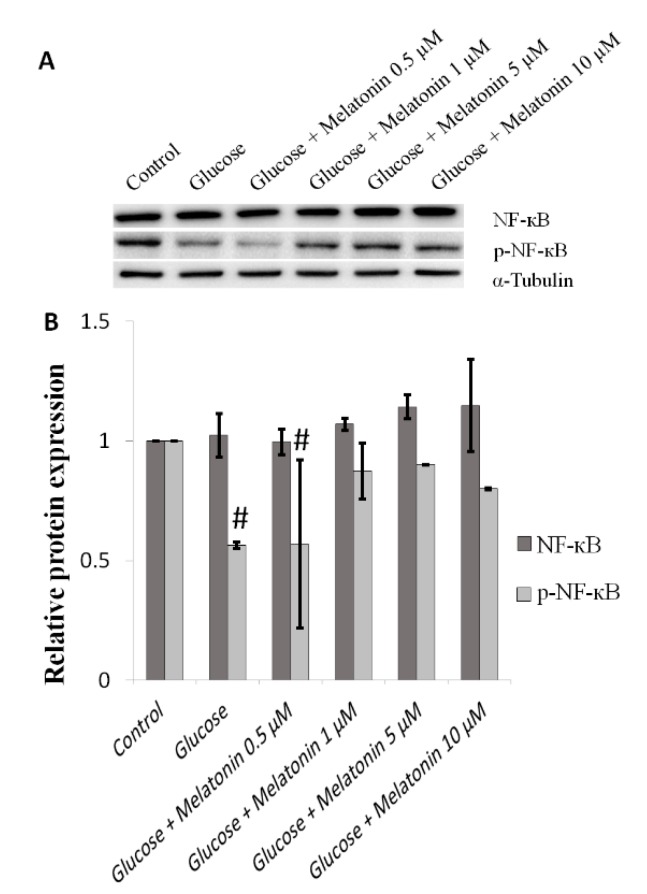
Effects of melatonin on NF-κB family proteins expression in high glucose-treated Schwann cells. The Schwann cells were treated with 100 mM of glucose with or without melatonin (0.5, 1, 5 and 10 μM) for 24 h. (**A**) represents NF-κB family proteins expression was measured by Western blot. Tubulin was used as the housekeeping protein. (**B**) is a bar chart of proteins expression; each bar represents mean ± SD of three independent experiments. * *p* < 0.05 significant different vs. 100 mM glucose; # *p* < 0.05 vs. control.

The effects of melatonin on NF-κB proteins expression in high glucose-treated Schwann cells was measured by Western blot. NF-κB has been reported to stimulate cell survival and reduction in its activity resulted in apoptosis [23,24,25]. Our results showed that there are no significant changes of NF-κB protein expression in high glucose-treated cells with or without melatonin treatment (Figure 6A,B). The expression level of p-NF-κB protein was downregulated in high glucose-treated cells. However, when high glucose-treated cells were treated with melatonin (0.5, 1, 5 and 10 μM), p-NF-κB protein expression significantly increased. This effect is especially prominent at concentrations of 5 µM and 10 µM of melatonin (Figure 6A,B).

### 3.7. Effects of Melatonin on mTOR Family Proteins Expression in High Glucose-Treated Schwann Cells

**Figure 7:**
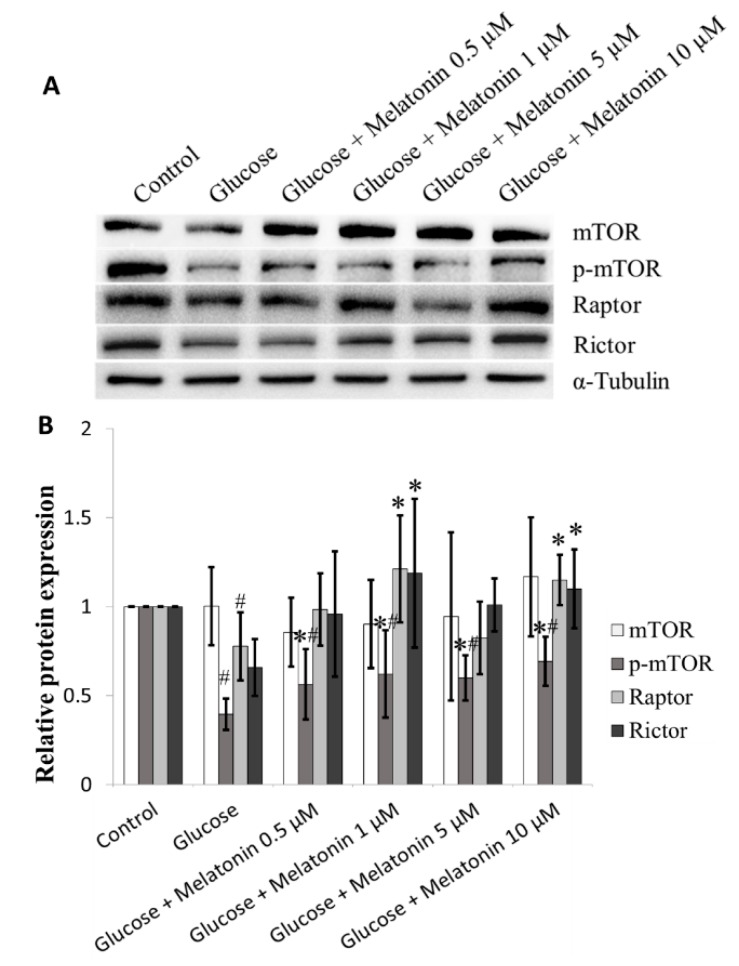
Effects of melatonin on mTOR family proteins expression in high glucose-treated Schwann cells. The Schwann cells were treated with 100 mM of glucose with or without melatonin (0.5, 1, 5 and 10 μM) for 24 h. (**A**) represents mTOR family proteins expression was measured by Western blot. Tubulin was used as the housekeeping protein. (**B**) is a bar chart of proteins expression; each bar represents mean ± SD of three independent experiments. * *p* < 0.05 significant different vs. 100 mM glucose; # *p* < 0.05 vs. control.

Our results found that high glucose down-regulate mTOR, p-mTOR, Rictor and Raptor proteins expression. Our result shows that melatonin (0.5, 1, 5 and 10 μM) treatment, significantly increased mTOR, p-mTOR, Rictor and Raptor proteins expression in high glucose-treated Schwann cells (Figure 7A,B).

### 3.8. Effects of Melatonin on Wnt Family Proteins Expression in High Glucose-Treated Schwann Cells

**Figure 8:**
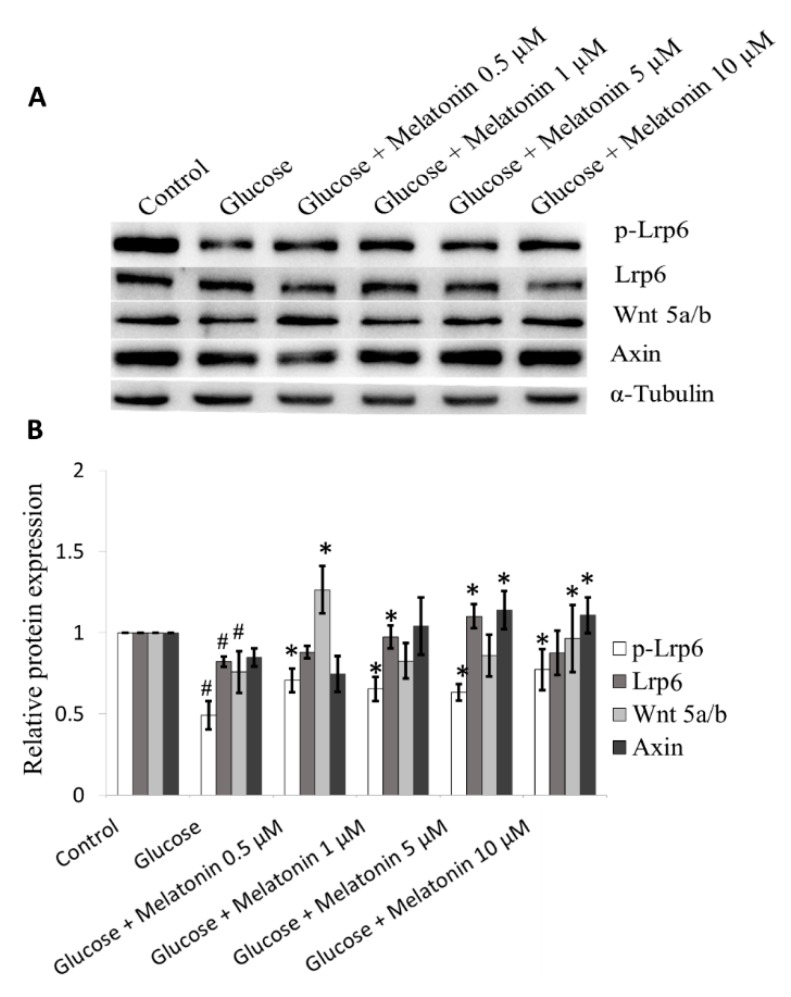
Effects of melatonin on Wnt family proteins expression in high glucose-treated Schwann cells. The Schwann cells were treated with 100 mM of glucose with or without melatonin (0.5, 1, 5 and 10 μM) for 24 h. (**A**) represents Wnt family proteins expression was measured by Western blot. Tubulin was used as the housekeeping protein. (**B**) is a bar chart of proteins expression; each bar represents mean ± SD of three independent experiments. * *p* < 0.05 significant different vs. 100 mM glucose; # *p* < 0.05 vs. control.

The effects of melatonin on Wnt family proteins expression in high glucose-treated Schwann cells was measured by Western blot. In Wnt signalling pathway activation, Wnt proteins bind to LRP, which signals Dv1 to inhibit constitutively active glycogen synthase kinase-3 beta. This causes nuclear translocation of b-catenin where it binds to the TCF4/lymphoid enhancer factor to form a transcriptional complex that regulates target genes translation that are involved in differentiation, proliferation, and apoptosis [26,27]. Our results demonstrated that high glucose down-regulates Wnt 5a/b, p-Lrp6, and Axin proteins expression. Melatonin treatment (0.5, 1, 5 and 10 μM) significantly increased Wnt 5a/b, p-Lrp6, and Axin proteins expression in high glucose-treated Schwann cells and the best effects were obtained at 5 and 10 µM melatonin (Figure 8A,B).

## 4. Discussion

In the present study, Schwann cells were treated with 100 mM glucose to mimic the hyperglycemic condition. Our data revealed that high glucose is cytotoxic to Schwann cells, and this is probably attributed to the multiple cytotoxic events including increased ROS levels, depolarisation of mitochondria and promotion of apoptosis. It is generally accepted that mitochondria are the primary source of endogenous ROS in mammalian cells. ROS are constantly generated through a mitochondrial electron transport chain as a by-product of ATP synthesis. In normal physiological conditions, ROS are scavenged by endogenous antioxidants such as glutathione and catalase to maintain oxidative homeostasis [28]. Oxidative stress occurs due to the imbalance of ROS generation and antioxidant activity. The accumulation of ROS, which creates an imbalance of intercellular redox state, is a common feature in diabetes [29,30]. Besides ROS-induced cytotoxicity, previous research also found that hyperglycemia can activate polyol pathway. Specifically, intracellular glucose is converted by aldose reductase (AR) to sorbitol, accumulation of sorbitol elevates osmatic pressure and results in cell damage [31]. In conclusion, hyperglycemia induces cytotoxicity through oxidative stress and sorbitol accumulation.

Our results revealed that the deleterious effects of high glucose on Schwann cells, such as increased ROS levels and mitochondrial depolarisation, as well as increased events of apoptosis, were diminished with melatonin treatment. Melatonin can inhibit production of ROS by maintaining the integrity of mitochondria, therefore reducing apoptosis. Antioxidant capacity of melatonin is superior to other antioxidants, which includes vitamin C, vitamin E and glutathione, and, therefore, it is more efficient in protecting cells from oxidative stress [32]. Moreover, melatonin is an amphiphilic molecule that is able to cross most physiological barriers and enters cell organelles which including membrane, nucleus and mitochondrion easily. This characteristic of melatonin allows it to be a promising free radical scavenger [33,34]. Melatonin has been demonstrated to inhibit oxidative/nitrosative stress-induced mitochondrial dysfunction in neurodegenerative disease models including Parkinson’s and Alzheimer’s diseases [26]. Studies showed that melatonin prevents mitochondrial dysfunction through several mechanisms, including the reduction of oxidative stress, maintenance of MMP, inhibition of the release of cytochrome c and upregulation of anti-apoptotic mitochondrial protein such as Bcl-2 [35,36,37]. Notably, Schwann cells are especially vulnerable to oxidative stress because of their large population of mitochondrion, and mitochondrion are both the source and target of ROS [38,39]. Moreover, previous studies reported that melatonin is able to enhance neural stem cell and Schwann cell proliferation [40,41]. In consistent with these findings, our results found that cell viability increased more than 100% after treated with glucose and different concentrations of melatonin (Figure 1). Collectively, melatonin prevents high glucose-induced cell death through enhanced cell proliferation and its anti-oxidant property.

The possible mechanisms by which melatonin prevents high glucose-induced cell apoptosis is discussed below. Accumulating evidence found that melatonin’s protective actions are mediated through the membrane-bound MT1 or/and MT2 receptor on Schwann cells. Melatonin binds to MT1 and MT2 receptors and further activates different cellular responses [42,43]. MT1 receptor is a pertussis toxin-sensitive guanine nucleotide-binding protein that mediates the inhibition of adenylyl cyclase in local tissues [44]. On the other hand, MT2 receptor is a G protein-coupled receptor and is involved in inhibiting adenylyl cyclase and soluble guanylyl cyclase pathways [45]. Both melatonin receptors MT1 and MT2 have been shown to activate the phospholipase C pathway, followed by increase in the levels of inositol triphosphate (IP3) and 1,2-diacylglycerol (DAG), which are linked to the pertussis toxin-insensitive Gq protein [46]. Activation of IP3 stimulates flow of calcium ions (Ca^2+^) into pancreatic β-cells, resulting in insulin secretion [47]. On the other hand, melatonin activates PI3K/Akt pathway. Activated Akt stimulates mTOR phosphorylation and NF-κB activation, which are the central regulators of cell metabolism, growth, proliferation and survival. Additionally, melatonin reduces oxidative stress and results in expression of Bcl-2 and Bax as well as suppression of caspase-dependent apoptosis [48,49,50]. Importantly, our data revealed that melatonin promotes upregulation of anti-apoptotic proteins (Bcl-2, Bcl-xL and Mcl-1) expression, as well as downregulation of pro-apoptotic proteins (Bad and Puma) expression. Anti-apoptotic Bcl-2 proteins preserve survival by binding and inhibiting the pro-apoptotic Bcl-2 proteins. In contrast, Bad and Puma are responsible for acting as sensitisers to bind and displace activator BH3-only proteins by binding to anti-apoptotic Bcl-2 proteins, allowing effector proteins Bax and Bak to inhibit apoptosis [51]. It has been reported that upregulation of Bcl-2, Mc-1 and A1 anti-apoptotic proteins inhibit apoptosis in T cells, B cells and dendritic cells [52].

The results of this study also demonstrated that melatonin prevents high glucose-induced apoptosis through NF-κB expression. NF-κB plays a crucial role in cell proliferation, survival and differentiation [53]. NF-κB in its activated form, p-NF-κB was upregulated in Schwann cells treated with melatonin under a high glucose condition. This demonstrated that melatonin prevents high glucose-induced apoptosis through activation of NF-κB pathway. Nerve injury studies showed that NF-κB activation is responsible for regulating the production of antioxidant enzymes such as mitochondrial superoxide dismutase (MnSOD) and inducible nitric oxide synthase (iNOS), therefore is an important molecule in maintaining oxidative homeostasis [54,55,56].

This study also found that mTOR signalling is one of the pathways involved in the protective mechanism of melatonin in high glucose-induced Schwann cell apoptosis. Our results demonstrated the elevation of phosphorylated mTOR, Rictor and Raptor proteins, suggesting involvements of both MTORC1 and MTORC2. mTOR signalling is responsible for regulation of cellular growth events including proliferation, differentiation, survival and metabolism [57,58]. Several studies have also revealed that high glucose-induced apoptosis is mediated by mTOR activation in various cells such as prostate and kidney cells [59,60,61]. Additionally, Zhu et al. reported that prolonged exposure to high glucose negatively regulated the mTOR pathway and led to apoptosis in Schwann cells [62]. Koh demonstrated that melatonin inhibits ischemic brain injury through activation of mTOR [63]. Together, these studies further support our findings that melatonin protects Schwann cells against high glucose-induced apoptosis mediated by activation of mTOR pathway.

Furthermore, the findings of this study also revealed that melatonin inhibits high glucose-induced apoptosis in Schwann cells through Wnt signalling pathway. Hyperglycemia is a negative regulator of Wnt pathway in Schwann cells survival. High glucose-induced apoptosis via downregulation of the Wnt pathway has been observed in different types of cells including mesangial cells [64], lymphocytic cells [65] and lung cells [66]. Our results showed that melatonin inhibits high glucose-induced apoptosis in Schwann cells via upregulation of Wnt5a/b and its co-receptor LRP5/6. The Wnt signalling pathway is important for cell proliferation, polarity and migration. Activation of Wnt/β-catenin promotes cell spreading and lamellipodia formation in Schwann cells, contributing to Schwann cell development [67]. Additionally, Shen et al. demonstrated that melatonin inhibits neural cell apoptosis after spinal cord injury via activation of Wnt/βcatenin [68]. Together, these studies support our findings that the protective mechanism of melatonin against high glucose-induced apoptosis is mediated by activation of the Wnt pathway in Schwann cells.

## 5. Conclusions

**Figure 9:**
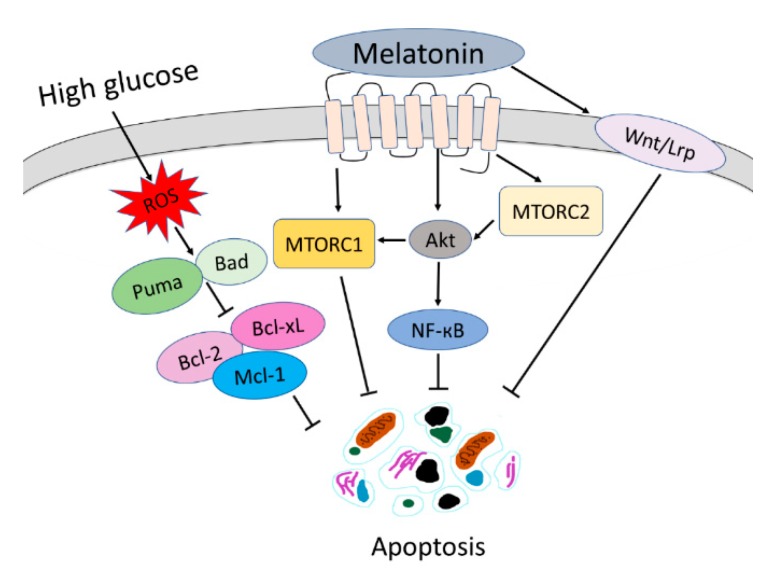
Summary of mechanisms of melatonin that protect high glucose-induced apoptosis in Schwann cells. MTORC1: mammalian target of rapamycin complex 1; MTORC2: mammalian target of rapamycin complex 2.

In conclusion, melatonin prevents high glucose-induced oxidative stress, depolarisation of MMP and apoptosis in Schwann cells. There are several signalling pathways involved in the protective mechanisms of melatonin against high glucose-induced apoptosis in Schwann cells, as summarised in Figure 9. Our finding proved that melatonin upregulates Bcl-2, Bcl-xL, Mc-1 and downregulates BAD and Puma to inhibit high glucose-induced apoptosis in Schwann cells. Besides, melatonin exhibited a protective effect against high glucose-induced apoptosis by promoting upregulation of the NF-κB, mTOR and Wnt signalling pathways in Schwann cells.

## References

[1] Deli G., Bosnyak E., Pusch A., Komoly S., Feher G. (2013). Diabetic neuropathies: Diagnosis and management. Neuroendocrinology.

[2] Farmer K.L., Li C., Dobrowsky R.T. (2012). Diabetic peripheral neuropathy: Should a chaperone accompany our therapeutic approach?. Pharm. Rev..

[3] Giacco F., Brownlee M. (2010). Oxidative stress and diabetic complications. Circ. Res..

[4] Gonçalves N.P., Vægte C.B., Andersen H., Østergaard L., Calcutt N.A., Jensen T.S. (2017). Schwann cell interactions with axons and microvessels in diabetic neuropathy. Neurology.

[5] Assaly R., de Tassigny A.D., Paradis S., Jacquin S., Berdeau A., Morin D. (2012). Oxidative stress, mitochondrial permeability transition pore opening and cell death during hypoxia-reoxygenation in adult cardiomyocytes. Eur. J. Pharmacol..

[6] Marchi S., Giorgi C., Suski J.M., Agnoletto C., Bononi A., Bonora M., De Marchi E., Missiroli S., Patergnani S., Poletti F. (2012). Mitochondria-ros crosstalk in the control of cell death and aging. J. Signal Transduct..

[7] Kuznetsov A.V., Margreiter R., Amberger A., Saks V., Grimm M. (2011). Changes in mitochondrial redox state, membrane potential and calcium precede mitochondrial dysfunction in doxorubicin-induced cell death. Biochim. Biophys. Acta.

[8] Hosseini A., Mohammad Abdollahi M. (2013). Diabetic neuropathy and oxidative Stress: Therapeutic perspectives. Oxidat. Med. Cell. Longev..

[9] Dan H.C., Cooper M.J., Cogswell P.C., Duncan J.A., Ting J.P., Baldwin A.S. (2008). Akt-dependent regulation of NF-κB is controlled by mTOR and Raptor in association with IKK. Genes Dev..

[10] Du Q., Geller D.A. (2010). Cross-Regulation between Wnt and NF-κB Signaling Pathways. Forum Immunopathol. Dis. Ther..

[11] Meng X., Li Y., Li S., Zhou Y., Gan R.Y., Xu D.P., Li H.B. (2017). Dietary Sources and Bioactivities of Melatonin. Nutrients.

[12] Choi D. (2013). Potency of melatonin in living beings. Dev. Reprod..

[13] Yu H., Dickson E.J., Jung S.R., Koh D.S., Hille B. (2016). High membrane permeability for melatonin. J. Gen. Physiol..

[14] Babaei-Balderlou F., Zare S., Heidari R., Farrokhi F. (2010). Effects of melatonin and vitamin E on peripheral neuropathic pain in streptozotocin-induced diabetic rats. Iran J. Basic Med. Sci..

[15] Brzezinski A. (1997). Melatonin in Humans. N. Engl. J. Med..

[16] Wang X., Figueroa B.E., Stavrovskaya I.G., Zhang Y., Sirianni A.C., Zhu S., Day A.L., Kristal B.S., Friedlander R.M. (2009). Methazolamide and melatonin inhibit mitochondrial cytochrome C release and are neuroprotective in experimental models of ischemic injury. Stroke.

[17] Ling X., Zhang L.M., Lu S.D., Li X.J., Sun F.Y. (1999). Protective effect of melatonin on injuried cerebral neurons is associated with bcl-2 protein over-expression. Zhongguo Yao Li Xue Bao.

[18] Koh P.O. (2008). Melatonin attenuates the focal cerebral ischemic injury by inhibiting the dissociation of pBad from 14-3-3. J. Pineal Res..

[19] Delaney C.L., Russell J.W., Cheng H.L., Feldman E.L. (2001). Insulin-like growth factor-I and over-expression of Bcl-xL prevent glucose-mediated apoptosis in Schwann cells. J. Neuropathol. Exp. Neurol..

[20] Li Y., Wu H., Liu N., Cao X., Yang Z., Lu B., Hu R., Wang X., Wen J. (2018). Melatonin exerts an inhibitory effect on insulin gene transcription via MTNR1B and the downstream Raf-1/ERK signaling pathway. Int. J. Mol. Med..

[21] Webster K.A. (2012). Mitochondrial membrane permeabilization and cell death during myocardial infarction: Roles of calcium and reactive oxygen species. Future Cardiol..

[22] Czabotar P.E., Lessene G., Strasser A., Adams J.M. (2014). Control of apoptosis by the BCL-2 protein family: Implications for physiology and therapy. Nat. Rev. Mol. Cell Biol..

[23] Mettang M., Reichel S.N., Lattke M., Palmer A., Abaei A., Rasche V., Huber-Lang M., Baumann B., Wirth T. (2018). IKK2/NF-κB signaling protects neurons after traumatic brain injury. FASEB J..

[24] Mincheva S., Garcera A., Gou-Fabregas M., Encinas M., Dolcet X., Soler R.M. (2011). The canonical nuclear factor-κB pathway regulates cell survival in a developmental model of spinal cord motoneurons. J. Neurosci..

[25] Imielski Y., Schwamborn J.C., Lüningschrör P., Heimann P., Holzberg M., Werner H., Leske O., Püschel A.W., Memet S., Heumann R. (2012). Regrowing the adult brain: NF-κB controls functional circuit formation and tissue homeostasis in the dentate gyrus. PLoS ONE.

[26] Marchetti B. (2018). Wnt/β-Catenin Signaling Pathway Governs a Full Program for Dopaminergic Neuron Survival, Neurorescue and Regeneration in the MPTP Mouse Model of Parkinson’s Disease. Int. J. Mol. Sci..

[27] Zarkou V., Galaras A., Giakountis A., Hatzis P. (2018). Crosstalk mechanisms between the WNT signaling pathway and long non-coding RNAs. Noncod. RNA Res..

[28] Victor V.M. (2014). Mitochondria oxidative stress in diabetes. Preedy VR. Diabetes-Oxidative Stress and Dietary Antioxidants.

[29] Asmat U., Abad K., Ismail K. (2016). Diabetes mellitus and oxidative stress—A concise review. Saudi Pharm. J..

[30] Volpe C.M.O., Villar-Delfino P.H., Dos Anjos P.M.F., Nogueira-Machado J.A. (2018). Cellular death, reactive oxygen species (ROS) and diabetic complications. Cell Death Dis..

[31] Hao W., Tashiro S., Hasegawa T., Sato Y., Kobayashi T., Tando T., Katsuyama E., Fujie A., Watanabe R., Morita M. (2015). Hyperglycemia promotes Schwann cell de-differentiation and de-myelination via sorbitol accumulation and Igf1 protein down-regulation. J. Biol. Chem..

[32] Túnez I., Muñoz M.C., Medina F.J., Salcedo M., Feijóo M., Montilla P. (2007). Comparison of melatonin, vitamin E and L-carnitine in the treatment of neuro- and hepatotoxicity induced by thioacetamide. Cell Biochem. Funct..

[33] Ramis M.R., Esteban S., Miralles A., Tan D.X., Reiter R.J. (2015). Protective effects of melatonin and mitochondria-targeted antioxidants against oxidative Stress: A Review. Curr. Med. Chem..

[34] Debnath B., Islam W., Min Li M., Sun Y., Lu X., Mitra S., Hussain M., Liu S., Qiu D. (2019). Melatonin mediates enhancement of stress tolerance in plants. Int. J. Mol. Sci..

[35] Srinivasan V., Spence D.W., Pandi-Perumal S.R., Brown G.M., Cardinali D.P. (2011). Melatonin in mitochondrial dysfunction and related disorders. Int. J. Alzheimers Dis..

[36] Zhao X.M., Hao H.S., Du W.H., Zhao S.J., Wang H.Y., Wang N., Wang D., Liu Y., Qin T., Zhu H.B. (2016). Melatonin inhibits apoptosis and improves the developmental potential of vitrified bovine oocytes. J. Pineal Res..

[37] Acuña Castroviejo D., López L.C., Escames G., López A., García J.A., Reiter R.J. (2011). Melatonin-mitochondria interplay in health and disease. Curr. Top. Med. Chem..

[38] Thorens B., Mueckler M. (2010). Glucose transporters in the 21st Century. Am. J. Physiol. Endocrinol. Metab..

[39] Wu Y., Xue B., Li X., Liu H. (2012). Puerarin prevents high glucose-induced apoptosis of Schwann cells by inhibiting oxidative stress. Neural Regen. Res..

[40] Li H., Zhang Y., Liu S., Li F., Wang B., Wang J., Cao L., Xia T., Yao Q., Chen H. (2019). Melatonin Enhances Proliferation and Modulates Differentiation of Neural Stem Cells Via Autophagy in Hyperglycemia: Mel Protects NSCs from Autophagy in HG. Stem Cells.

[41] Chang H.M., Liu C.H., Hsu W.M., Chen L.Y., Wang H.P., Wu T.H., Chen K.Y., Ho W.H., Liao W.C. (2014). Proliferative effects of melatonin on Schwann cells: Implication for nerve regeneration following peripheral nerve injury. J. Pineal Res..

[42] Ekmekcioglu C. (2006). Melatonin receptors in humans: Biological role and clinical relevance. Biomed. Pharmacother..

[43] Uz T., Arslan A.D., Kurtuncu M., Imbesi M., Akhisaroglu M., Dwivedi Y., Pandey G.N., Manev H. (2005). The regional and cellular expression profile of the melatonin receptor MT1 in the central dopaminergic system. Mol. Brain Res..

[44] Carlson L.L., Weaver D.R., Reppert S.M. (1989). Melatonin signal transduction in hamster brain: Inhibition of adenylyl cyclase by a pertussis toxin-sensitive g protein. Endocrinology.

[45] Petit L., Lacroix I., De Coppet P., Strosberg A.D., Jockers R. (1999). Differential signaling of human Mel1a and Mel1b melatonin receptors through the cyclic guanosine 3′-5′-monophosphate pathway. Biochem. Pharmacol..

[46] Jockers R., Maurice P., Boutin J.A., Delagrange P. (2008). Melatonin receptors, heterodimerization, signal transduction and binding sites: What’s new?. Br. J. Pharmacol..

[47] Brydon L., Roka F., Petit L., de Coppet P., Tissot M., Barrett P., Morgan P.J., Nanoff C., Strosberg A.D., Jockers R. (1999). Dual signalling of human Mel 1a melatonin receptors via G(i2), g(i3) and G(q/11) proteins. Mol. Endocrinol..

[48] Kim H.S., Kim T.J., Yoo Y.M. (2017). Melatonin combined with endoplasmic reticulum stress induces cell death via the PI3K/Akt/mTOR pathway in B16F10 melanoma cells. PLoS ONE.

[49] Kilic U., Caglayan A.B., Beker M.C., Gunal M.Y., Caglayan B., Yalcin E., Kelestemur T., Gundogdu R.Z., Yulug B., Yılmaz B. (2017). Particular phosphorylation of PI3K/Akt on Thr308 via PDK-1 and PTEN mediates melatonin’s neuroprotective activity after focal cerebral ischemia in mice. Redox Biol..

[50] Wang Y., Zeng S. (2018). Melatonin Promotes Ubiquitination of Phosphorylated Pro-Apoptotic Protein Bcl-2-Interacting Mediator of Cell Death-Extra Long (BimEL) in Porcine Granulosa Cells. Int. J. Mol. Sci..

[51] Serasinghe M.N., Missert D.J., Asciolla J.J., Podgrabinska S., Wieder S.Y., Izadmehr S., Belbin G., Skobe M., Chipuk J.E. (2015). Anti-apoptotic BCL-2 proteins govern cellular outcome following B-RAF(V600E) inhibition and can be targeted to reduce resistance. Oncogene.

[52] Carrington E.M., Zhan Y., Bray J.L., Zhang J.G., Sutherland R.M., Anstee N.S., Schenk R.L., Vikstrom I.B., Delconte R.B., Segal D. (2017). Anti-apoptotic proteins BCL-2, MC-1 and A1 summate collectively to maintain survival of immune cell populations both in vitro and in vivo. Cell Death Differ..

[53] Ruland J. (2011). Return to homeostasis: Downregulation of NF-κB responses. Nat. Immunol..

[54] Mariani E., Polidori M.C., Cherubini A., Mecocci P. (2005). Oxidative stress in brain aging, neurodegenerative and vascular diseases: An overview. J. Chromatogr. B Anal. Technol. Biomed. Life Sci..

[55] Sompol P., Xu Y., Ittarat W., Daosukho C., St Clair D. (2006). NF-kappaB-associated MnSOD induction protects against beta-amyloid-induced neuronal apoptosis. J. Mol. Neurosci..

[56] Ji L.L., Gomez-Cabrera M.C., Vina J. (2007). Role of nuclear factor kappaB and mitogen-activated protein kinase signaling in exercise-induced antioxidant enzyme adaptation. Appl. Physiol. Nutr. Metab..

[57] Engelman J.A. (2009). Targeting PI3K signalling in cancer: Opportunities, challenges and limitations. Nat. Rev. Cancer.

[58] Schmelzle T., Hall M.N. (2000). mTOR, a central controller of cell growth. Cell.

[59] Selvaraj S., Sun Y., Sukumaran P., Singh B.B. (2016). Resveratrol activates autophagic cell death in prostate cancer cells via downregulation of STIM1 and the mTOR pathway. Mol. Carcinog..

[60] Geuna E., Roda D., Rafii S., Jimenez B., Capelan M., Rihawi K., Montemurro F., Yap T.A., Kaye S.B., De Bono J.S. (2015). Complications of hyperglycemia with PI3K-AKT-mTOR inhibitors in patients with advanced solid tumours on Phase 1 clinical trials. Br. J. Cancer.

[61] Crouthamel M.C., Kahana J.A., Korenchuk S., Zhang S.Y., Sundaresan G., Eberwein D.J., Brown K.K., Kumar R. (2009). Mechanism and management of AKT inhibitor-induce hyperglycemia. Clin. Cancer Res..

[62] Zhu L., Hao J., Cheng M., Zhang C., Huo C., Liu Y., Du W., Zhang X. (2018). Hyperglycemia-induced Bcl-Bax-mediated apoptosis of Schwann cells via mTORC1/S6K1 inhibition in diabetic peripheral neuropathy. Exp. Cell Res..

[63] Koh P.O. (2008). Melatonin prevent ischemic brain injury through activation of the mTOR/p70S6 kinase signaling pathway. Neurosci. Lett..

[64] Lin C.L., Wang J.Y., Huang Y.T., Kuo Y.H., Surendran K., Wang F.S. (2006). Wnt/β-catenin signaling modulates survival of high glucose-stressed mesangial cells. Pathophysiol. Ren. Dis. Prog..

[65] Lu D., Choi M.Y., Yu J., Castro J.E., Kipps T.J., Carson D.A. (2011). Salinomycin inhibits Wnt signalig and selectively induces apoptosis in chronic lymphocytic leukaemia cells. Proc. Natl. Acad. Sci. USA.

[66] Lee J.S., Hur M.W., Lee S.K., Choi W.L., Kwon Y.G., Yun C.O. (2012). A novel sLRP6E1E2 inhibits conical Wnt signaling, epithelial-to-mesenchymal transition, and induces mitochondria-dependent apoptosis in lung cancer. PLoS ONE.

[67] Grigoryan T., Stein S., Qi J., Wende H., Garrratt A.N., Nave K.A., Birchmeier C., Birchmeier W. (2013). Wnt/Rspondin/β-catenin signals control axonal sorting and lineage progression in Schwann cell development. Proc. Natl. Acad. Sci. USA.

[68] Shen Z., Zhou Z., Gao S., Guo Y., Gao K., Wang H., Dang X. (2017). Melatonin inhibits neural cell apoptosis and promotes locomotor recovery via activation of Wnt/β-catenin signaling pathway after spinal cord injury. Neurochem. Res..

